# Use of Surgical Adhesive Tape to Maintain Tension on Intrafocal K-Wires for Easy Reduction and Fixation of Complex Intra-Articular Distal Radius Fractures

**DOI:** 10.7759/cureus.12427

**Published:** 2021-01-02

**Authors:** Jonathan M French, Angelos Assiotis, Harpal S Uppal

**Affiliations:** 1 Trauma and Orthopaedics, Bristol Royal Infirmary, Bristol, GBR; 2 Trauma and Orthopaedics, Lister Hospital, Stevenage, GBR

**Keywords:** distal radius fracture, intrafocal, reduction, fixation

## Abstract

Maintaining reduction during fixation of complex intra-articular distal radius fractures with dorsal comminution can be challenging. We describe an operative technique where reduction is achieved with temporary intrafocal Kirschner wires (K-wires), and held using surgical adhesive tape wrapped around the hand, whilst a volar plate is applied to achieve rigid fixation. This is a simple, inexpensive method used at our institutions which allows fixation of these fractures without the need for an operative assistant.

## Introduction

Distal radius fractures with dorsal angulation, comminution, and/or intra-articular extension are often challenging injuries to treat. They can be managed operatively with a volar locking plate, or column-specific plates through dorsal or radial approaches. If a single volar plate is used, maintaining adequate reduction of the fragments intraoperatively can be difficult. The use of intrafocal Kirschner wires (K-wires) to aid internal fixation of the distal radius has previously been described in the literature [1-3]. The premise of the technique is that a percutaneous K-wire is inserted intrafocally to lever the distal fragment to a reduced position, similarly to the Kapandji method [4]. These wires are temporary and aim to maintain satisfactory reduction whilst the distal radius plate is applied.

Here we present a case to demonstrate our technique that builds on the above, with novel use of equipment. It is a simple, cost-effective method allowing reduction and fixation of complex distal radius fractures with dorsal displacement and comminution, with or without an intra-articular component, by a single surgeon.

## Technical report

Example case report

**Figure 1:**
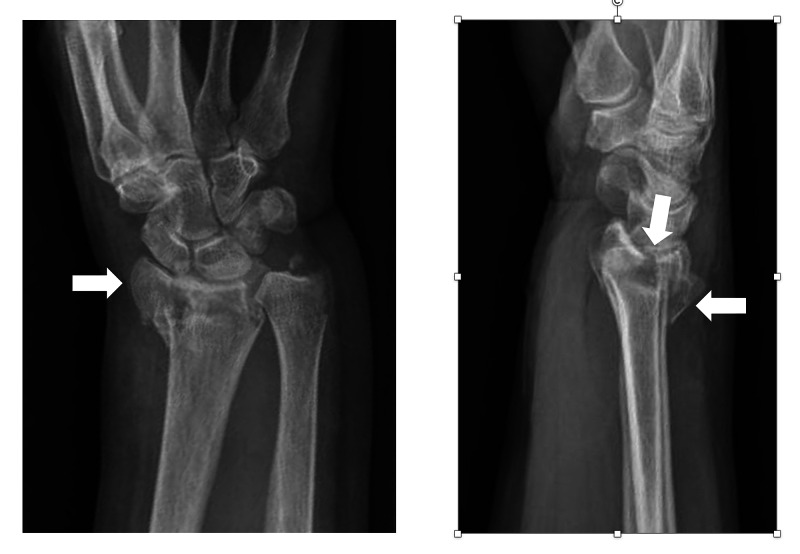
Anteroposterior (AP) and lateral plain radiographs of the right wrist showing initial injury White arrows highlight (from left to right) the separate radial styloid fragment, intra-articular split, and dorsal comminution.

A 72-year-old female injured her right dominant wrist after falling onto her outstretched hand from her bicycle. She noted immediate pain and an obvious deformity of the wrist, and presented to our emergency department. Clinical examination indicated a closed injury with normal function of the median nerve in the hand. She then underwent imaging with plain radiographs and an attempt at closed reduction under regional anaesthesia (Figure 1). Subsequent radiographs and computed tomography (CT) imaging showed a dorsally displaced, intra-articular fracture with dorsal comminution and a separate radial styloid fragment. There was also an ulnar styloid fracture. At the time of injury, she was otherwise healthy and not taking any regular medications. She did not smoke or consume alcohol. The decision was made to proceed to operative fixation in order to restore radial height, inclination, and volar tilt. The operative technique is described below.

**Figure 2:**
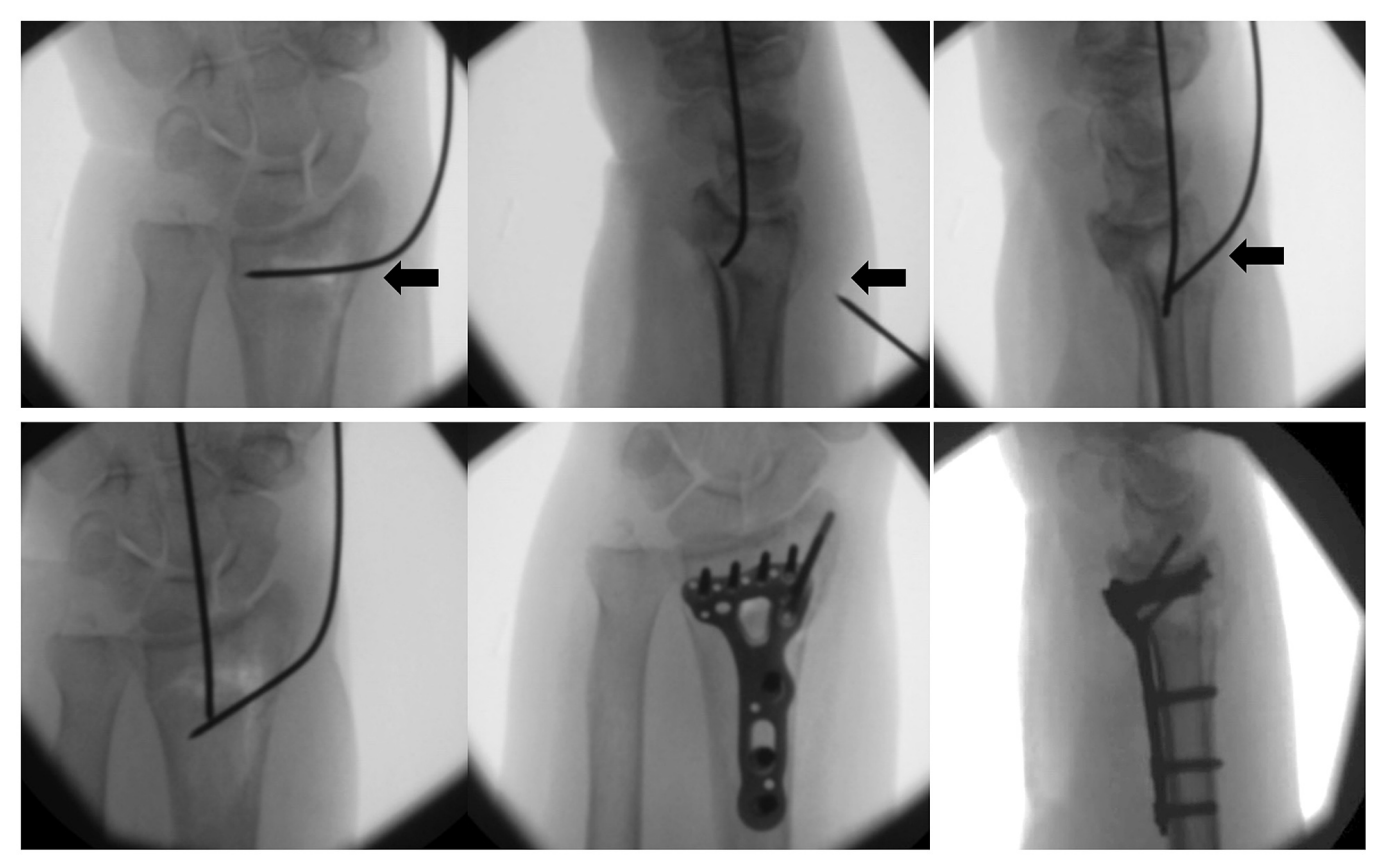
Intraoperative fluoroscopic image sequence (PA) demonstrating the reduction technique employing two 1.6 mm K-wires, bent and held with adhesive surgical tape, in order from left to right, top then bottom Black arrows highlight intrafocal K-wire insertion. Reduction is seen after the wires are bent distally and approximated to the hand.

**Figure 3:**
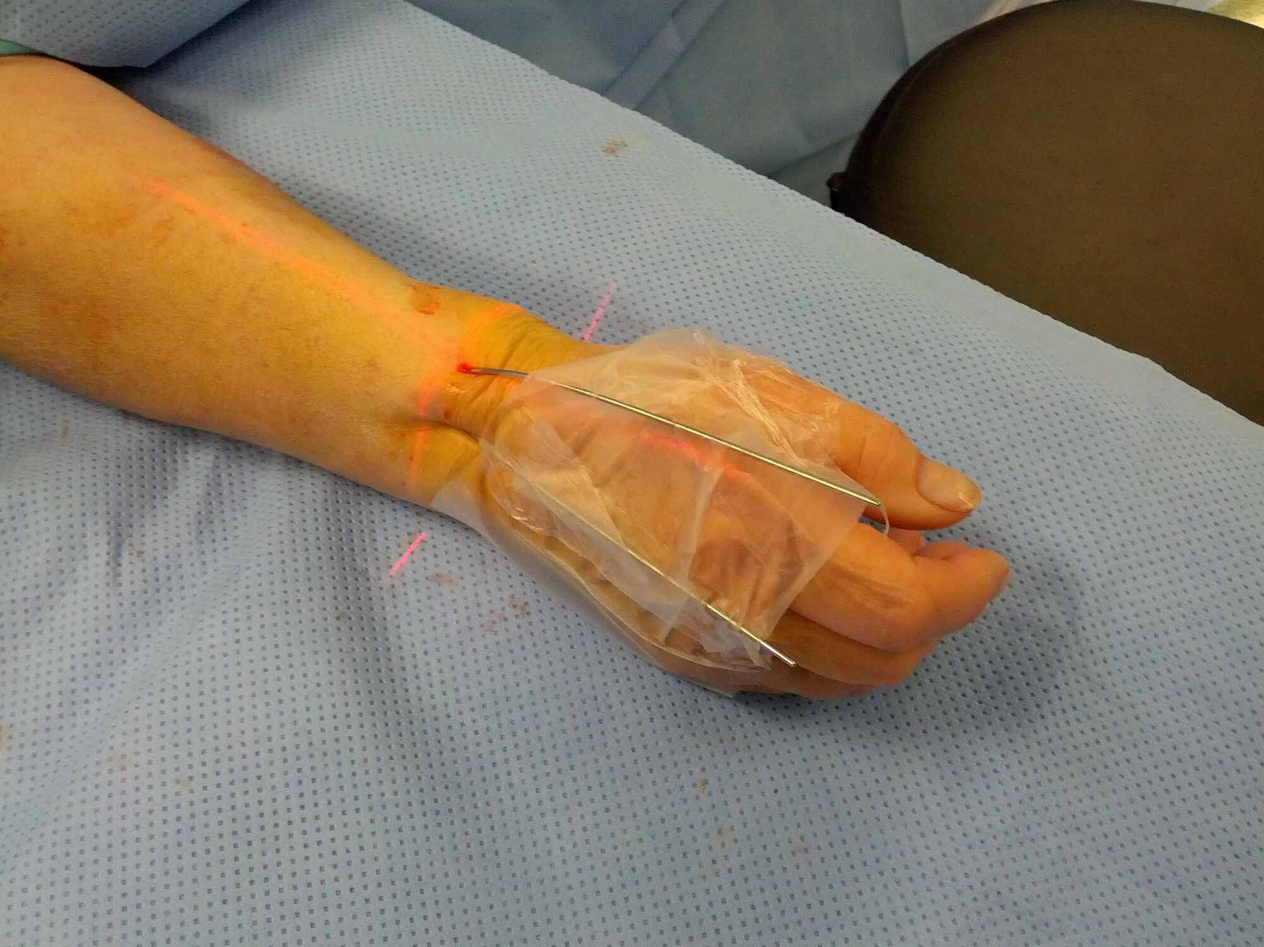
Clinical photograph demonstrating construct on the patient’s hand

We routinely use an flexor carpi radialis (FCR)-bed approach to the volar distal radius, with brachioradialis release for all displaced radial styloid fragment fracture patterns. Following the approach, the reduction is performed by percutaneously inserting 1.6 mm intrafocal K-wires under image intensifier control, from the fracture site to the intramedullary canal. These are held with adhesive tape, to stabilise the fracture by buttressing the dorsal and radial cortices whilst applying the volar locking plate. The wires are placed entirely by hand, without power, to avoid damage to dorsal and radial structures. They should not engage the opposite cortex as this would not permit manual reduction. The wires are pushed through the radial and dorsal skin, then rotated and bent distally to correct the radial and volar tilt of the distal fragments (Figure 2). Once adequate reduction has been achieved, the wires are approximated to the patient’s hand. A strip of sterile adhesive surgical tape is then wrapped tightly around the hand, including the wire, in order to maintain tension and therefore the reducing forces of the two pre-loaded wires (Figure 3). Use of 1.6 mm diameter wires is key to ensure the correct amount of tension whilst still allowing them to be bent.

The order of wire insertion is left to the discretion of the surgeon, but in our practice, we routinely reduce the styloid fragment first, apply the first wrap of adhesive tape to the hand, and then reduce the dorsal tilt. This construct is stable enough that the hand can be rotated freely and placed flat on the table, without interference from the protruding wire ends, and without the need for an assistant in order to maintain reduction. The reduction can easily be checked on image intensifier, and further fragment-specific wires added if necessary, for example to reduce dorsal lunate fragments. The volar locking plate is then placed to definitively hold the reduction, without interference from the dorsal wires. The wires are subsequently removed and stability assessed on the table.

## Discussion

We routinely employ this technique to manage complex dorsally angulated distal radius fractures at our institutions. In 2010, Stohr et al. described the use of intrafocal K-wires as a lever to reduce and temporarily stabilise intra-articular distal radius fractures with dorsal comminution and angulation [1]. More recent studies describe a similar technique using straight, retrograde intrafocal wires, with the addition of a radial wire to correct height and inclination, to temporarily hold reduction whilst a volar plate is applied [2,3]. The advantages our technique has are: firstly, the use of surgical tape maintains the tension on the construct to hold reduction, and prevents the wires from impinging on the drapes. Secondly, as the tension is held by the surgical tape, it is not routinely necessary to have a surgical assistant. Thirdly, wires are placed by hand rather than on power, minimising the risk of avulsion injuries to neurovascular structures, in particular, the superficial radial nerve (SRN) and the extensor compartment tendons; in the past few years, we have used this technique in an excess of one hundred cases with no incidence of damage to the SRN or tendons. Fourthly, as all wires are either dorsal or radial, and intrafocal, they do not impede plate or screw placement. Finally, additional wires can be used for fragment-specific reduction; additional pieces of surgical tape can be added on top of the previous ones.

## Conclusions

This technique is therefore a versatile, cost-effective, reproducible method for reducing and stabilising complex intra-articular distal radius fractures with dorsal comminution and displacement.
